# Autoimmune Regulator Expression in DC2.4 Cells Regulates the NF-κB Signaling and Cytokine Expression of the Toll-Like Receptor 3 Pathway

**DOI:** 10.3390/ijms17122002

**Published:** 2016-12-01

**Authors:** Jitong Sun, Kunwei Niu, Haiying Fu, Haijun Li, Yi Li, Wei Yang

**Affiliations:** Department of Immunology, College of Basic Medical Sciences, Jilin University, Changchun 130021, China; sjitong@163.com (J.S.); niukw123@163.com (K.N.); fuhy@jlu.edu.cn (H.F.); lidongbei2008@163.com (H.L.)

**Keywords:** autoimmune regulator, immune tolerance, TLR3

## Abstract

Autoimmune regulator (Aire) mutations result in autoimmune polyendocrinopathy candidiasis ectodermal dystrophy (APECED), which manifests as multi-organ autoimmunity and chronic mucocutaneous candidiasis (CMC). Indendritic cells (DCs), pattern recognition receptors (PRR), such as Toll-like receptors (TLRs), are closely involved in the recognition of various pathogens, activating the intercellular signaling pathway, followed by the activation of transcription factors and the expression of downstream genes, which take part in mediating the immune response and maintaining immune tolerance. In this study, we found that Aire up-regulated TLR3 expression and modulated the downstream cytokine expression and nuclear factor-κB (NF-κB) of the TLR3 signaling pathway.

## 1. Introduction

Toll-like receptors (TLRs), which are mainly expressed in antigen-presenting cells (APCs) such as macrophages and dendritic cells (DCs), serve as pattern recognition receptors (PRRs), recognize a wide range of pathogen-associated molecular patterns (PAMPs) from diverse pathogens, including bacteria, viruses, and fungi. These receptors play a central role in the initiation of innate immune responses. Additionally, TLRs can also recognize and eliminate self-senescent and pathological cells to maintain immune homeostasis [1]. Mammalian TLRs comprise a large family consisting of 13 members. Eleven of them are expressed in human cells [2]. On DCs, TLR3 expression permits the recognition of *Candida* yeasts, as well as some self-components, and activates the intercellular TLR adaptor molecule 1 (TICAM-1 or TRIF)-dependent signaling pathway, which subsequently activates transcription factors, such as interferon regulatory factor 3 (IRF-3) and nuclear factor-κB (NF-κB). Additionally, TLR3 expression induces the expression of type I interferon (type I IFN), interleukin 23 (IL-23), and IL-27, as well as T cell stimulatory molecules, MHC class II, CD40, CD80, CD83, and CD86, taking part in mediating the immune response and maintaining immune tolerance [3,4,5,6]. However, the regulation mechanism of TLR3 and its pathway is still unclear.

Autoimmune regulator (Aire), as a transcript factor, can regulate the expression of thousands of genes, and plays a very important role in the maintenance of immune tolerance and the defense of pathogenic infection [7]. Single genetic mutation of Aire leads to Autoimmune polyendocrinopathy candidiasis ectodermal dystrophy (APECED), also known as autoimmune polyendocrine syndrome type I (APS-1). The typical symptoms of APECED include hypoparathyroidism, primary adrenocortical failure, and chronic mucocutaneous candidiasis (CMC). Most APECED patients also exhibit other autoimmune diseases and deficiencies, including thyroiditis, type 1 diabetes (T1D), or autoimmune hepatitis [8]. Aire is mainly expressed in the medullary thymic epithelial cells (mTECs). It is essential to prevent autoimmune diseases by inducing the expression of a broad repertoire of tissue-restricted antigens (TRAs) and promoting the clonal deletion of self-reactive thymocytes [9]. Gardner et al. [9] reported that extrathymic Aire-expressing cells (eTAC) were bone marrow-derived peripheral antigen-presenting cells (APC) that could functionally inactivate CD4^+^ T cells. The eTAC express some of the same markers as conventional DCs along with TRAs, but comprise a tolerogenic population distinct from conventional DCs [10,11]. In our previous study, we found that Aire-overexpressing macrophages could induce increases in the numbers of CD4^+^CD45RA^+^Foxp3^hi^ and CD4^+^CD45RA^−^Foxp3^hi^ activating Tregs (aTreg) [12]. We also found that the TLR1, TLR3, and TLR8 expression levels were up-regulated in Aire-overexpressing murine macrophages [13,14]. Therefore, we hypothesized that Aire expressed in DCs could regulate TLR3 expression and influence the activation of TLR3 intracellular signaling, to regulate immune response and tolerance.

In this study, we established a stable Aire-expressed mouse DC cell line, with up-regulated TLR3 expression. Aire could also influence TLR3 pathway-mediated cytokine production, as well as the NF-κB signaling pathway, therefore regulating the immune response in the steady state and maintain the tolerance under inflammation.

## 2. Results

### 2.1. Establishment of Stable Aire-Expressing Transfectants (Stable Aire Expression in DC2.4 Cells)

**Figure 1:**
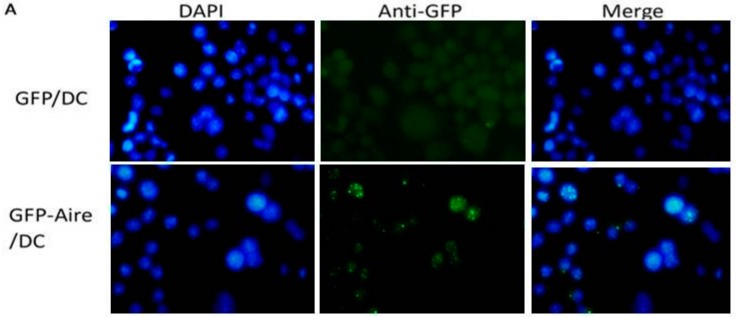

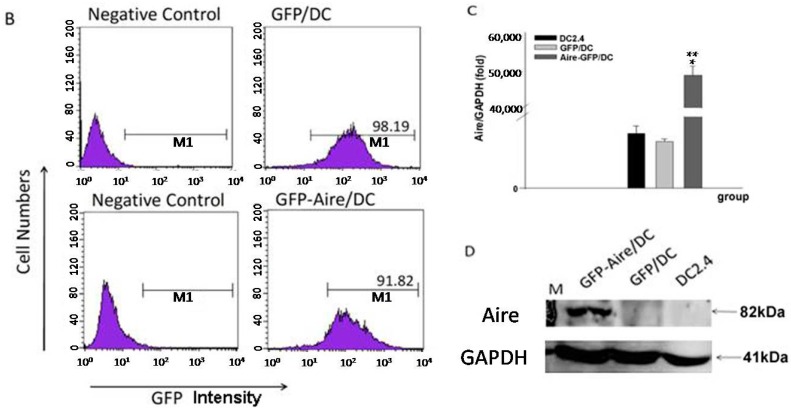
Identification of stable Aire-expressing transfectants. (**A**) Observation of stably-transfected cells. DC2.4 cells were transfected with pEGFPC1/Aire or pEGFPC1 expression vectors and selected with G418 as previously described. G418-selected cells were observed via fluorescence microscopy (GFP positive cells are shown as green dots). Nuclei were detected by DAPI staining (blue). Original magnification, 400×; (**B**) The transfection efficiency was assessed via FCM (the GFP positive cells were gated with M1); and (**C**,**D**) The Aire mRNA and protein expression levels were determined by real-time PCR and Western blotting analyses. * *p* < 0.01 vs. DC2.4; ** *p* < 0.01 vs. GFP/DC.

Aire is expressed at a low level in peripheral hematopoietic cells such as DCs. To study the roles and mechanisms of Aire in DCs, we transfected a Aire vector that we had previously constructed and pEGFPC1 as a control into DC2.4 cells, and obtained stably-transfected DC2.4 cells that expressed the fusion protein (GFP-Aire/DC) or GFP alone (GFP/DC). As shown in Figure 1A, GFP-Aire was expressed primarily in discrete speckles representing the nuclear bodies (NBs), consistent with the localization of endogenous Aire reported in a former study. The percentages of GFP positive clones of GFP-Aire/DC and GFP/DC were 91.82% and 98.19%, respectively (Figure 1B). At the mRNA level, Aire expression in GFP-Aire/DC was 37.8-fold higher than that in GFP/DC (Figure 1C); similar results were observed at the protein level (Figure 1D). Thus, we demonstrated that the GFP-Aire fusion protein was specifically expressed in stably-transfected DC2.4 cells.

### 2.2. Aire Regulates TLR Expression in Stable Aire-Expressing DC2.4 Cells

**Figure 2:**
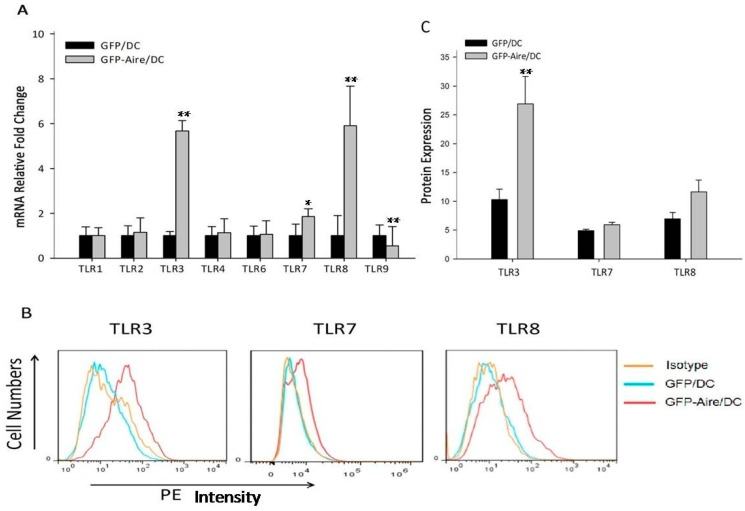
The effects of Aire on TLR expression in GFP-Aire/DC. (**A**) The levels of TLR1–9 transcript expression in GFP-Aire/DC and GFP/DC were detected by RT-qPCR. All qPCR data are shown as the gene expression relative to GAPDH and are depicted as fold changes relative to the expression in GFP/DC cells, which was normalized to 1; (**B**) The TLR1, TLR3, and TLR8 protein expression levels in stably-transfected DC2.4 cells were analyzed by FCM. The proteins were detected with anti-TLR3, anti-TLR7, and anti-TLR8 antibodies, respectively, as well as a PE-conjugated goat anti-rabbit IgG; and (**C**) The bar graph depicts the expression levels of TLR3, TLR7, and TLR8 according to the MFI values. Data are shown as the means ± SD from three to six independent experiments. GFP-Aire/DC vs. GFP/DC: * *p* < 0.05; ** *p* < 0.01.

To confirm whether Aire could regulate TLR expression in DCs, we assessed the TLR expression levels in Aire-GFP/DC and GFP/DC. The results revealed that TLR3 (5.7-fold ± 0.4) and TLR8 (5.9-fold ± 1.7) in particular, as well as TLR7 (1.8-fold ± 0.3), were notably up-regulated in the GFP-Aire/DC cells. Additionally, TLR9 was downregulated in the GFP-Aire/DC cells (0.5-fold ± 0.9). No significant differences in TLR1, TLR2, TLR4, and TLR6 expression were observed between the GFP-Aire/DC and GFP/DC (Figure 2A). We failed to determine the TLR5 expression level in DC2.4. TLR3 protein expression was also up-regulated, which was consistent with the above-described results, but no significant differences were observed with respect to TLR7 and TLR8 protein expression (Figure 2B,C). Therefore, these data indicate that Aire potentially regulates TLR3 expression in DCs.

### 2.3. Aire Influences the Phenotype of DC2.4 Cells

The exposure of BMDCs to TLR3 agonists has been reported to induce co-stimulatory molecule up-regulation and cell maturation. To determine whether Aire could influence the phenotype of DCs, we stimulated GFP-Aire/DC and GFP/DC with the TLR3 ligand Poly (I:C) as described previously, and analyzed the CD40, CD80, CD83, CD86, CD11C, and MHC II expression levels using FCM. The results revealed that both GFP/DC and GFP-Aire/DC expressed low levels of CD40, CD86, and MHCII. In the untreated cells, CD83, CD11C, and particularly CD80 expression were decreased in GFP-Aire/DC, compared with GFP/DC cells. When Poly (I:C) was added, these decreases in expression were amplified (Table 1). These findings indicated that Aire might delay Poly (I:C)-induced DC maturation.

### 2.4. Aire Regulates Cytokine Expression Downstream of the TLR3 Pathway

**Figure 3:**
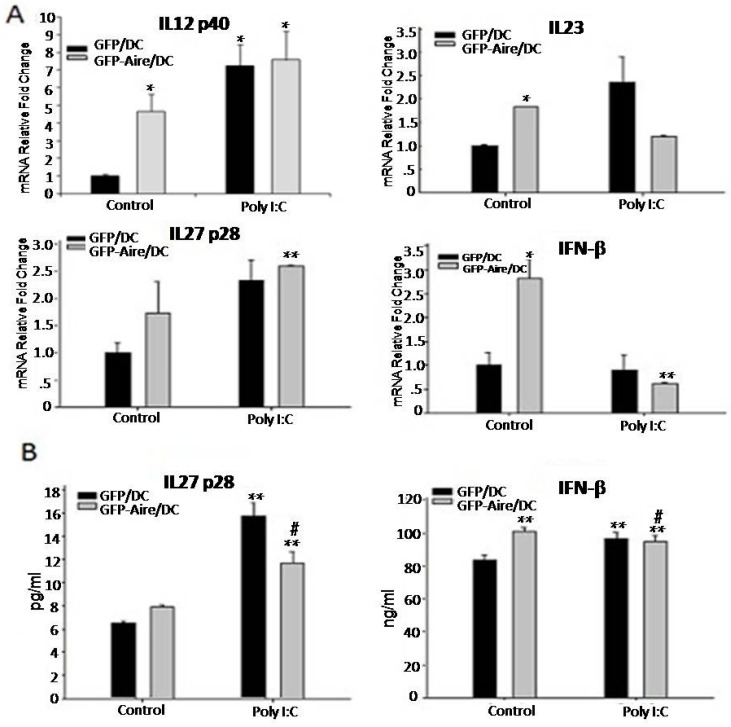
The effects of Aire on gene expression downstream of TLR3 in GFP-Aire/DC. (**A**) The IL-12 p40, IL-23, IL-27 p28, and IFN-β transcript expression levels in GFP-Aire/DC and GFP/DC cells were detected by RT-qPCR. All qPCR data are shown as the expression relative to that of GAPDH and are depicted as fold-change relative to the expression in GFP/DC cells, which was normalized to 1; and (**B**) The IL-27 p28 and IFN-β protein concentrations in GFP-Aire/DC and GFP/DC cells were analyzed by ELISA. Data are shown as the means ± SD from three to six independent experiments. * *p* < 0.05 and ** *p* < 0.01 compared with the GFP/DC control; # *p* < 0.05 compared with the GFP-Aire/DC control.

Based on the previous results, which demonstrated that Aire could regulate TLR3 expression and influence the phenotype of DC2.4 cells following TLR3 pathway activation, we next evaluated the transcription of other genes downstream of TLR3 such as IL-12p40, IL-23, IL-27p28, and IFN-β in Poly (I:C)-treated GFP-Aire/DC and GFP/DC cells. As shown in Figure 3A, IL-12p40, IL-23, and IFN-β expression were increased in the untreated GFP-Aire/DC. The IFN-β expression was decreased in the Poly (I:C)-treated GFP-Aire/DC than those untreated GFP-Aire/DC, which suggested that Aire might promote IFN-β expression in a TLR3-independent manner, or TLR3 stimulation interfering with the function of Aire on type 1 IFN regulator (IRF), IRF3, or IRF7. However, no differences were observed between Poly (I:C)-treated and untreated GFP-Aire/DC. The results from lower-dose Poly (I:C)-treated cells were consistent with those of the former experiment (Figure S1). Additionally, changes in the IL-27p28 and IFN-β protein levels were consistent with those at the mRNA level (Figure 3B). However, the IL-12p40 and IL-23 concentrations in our samples were below the detection limit. Therefore, the mechanism by which Aire regulates gene expression downstream of TLR3 will require further study.

### 2.5. Aire Modifies Key TLR3 Pathway Molecules in DC2.4

**Figure 4:**
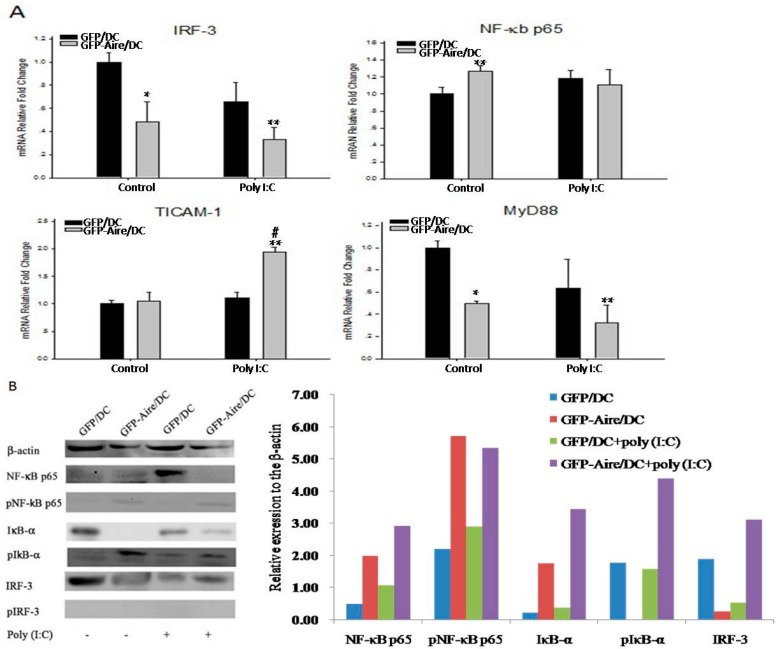

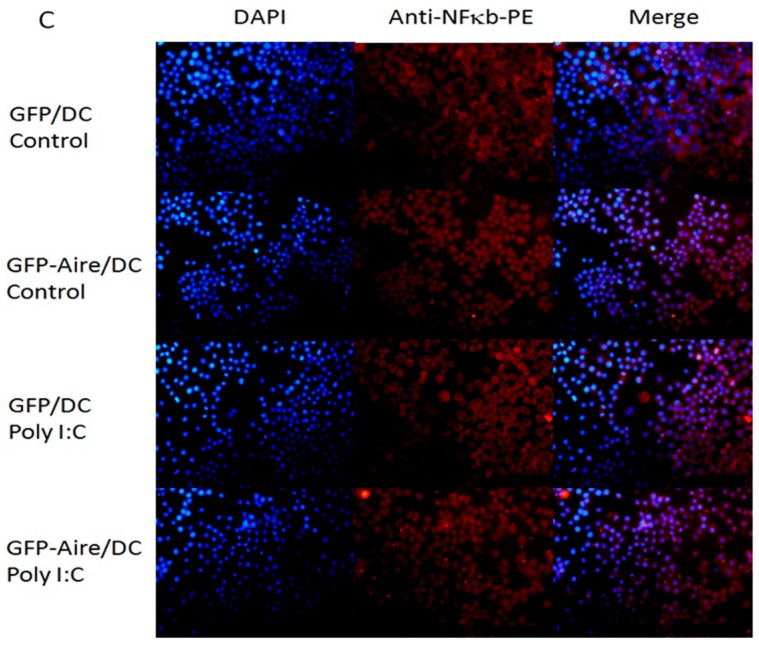
Aire modifies key TLR3 pathway molecules in DC2.4 cells. (**A**) The IRF-3, NF-κB, TICAM-1, and MyD88 transcript expression levels were detected in GFP-Aire/DC and GFP/DC by RT-qPCR. All qPCR data are shown as the expression calculated relative to that of GAPDH and are depicted as fold changes relative to the expression in GFP/DC cells, which was normalized to 1; (**B**) NF-κB, IκB-α, and IRF-3 protein expression levels were measured by Western blotting. The histograms are the relative expression of each molecules compared to the β-actin; and (**C**) Immunofluorescent detection of NF-κB (**red**) translocation to the nuclei (**blue**; combined signal, **purple**) in DC2.4 cells. Original magnification, 200×. A representative experiment of three independent experiments is shown. Data are shown as the means ± SD from three to six independent experiments. * *p* < 0.05 and ** *p* < 0.01 compared with the GFP/DC control; # *p* < 0.05 compared with the GFP-Aire/DC control.

Having demonstrated that Aire influenced the expression of TLRs and downstream cytokines, we next attempted to determine the key TLR3 pathway molecules responsible for these effects. The RT-qPCR showed that only NF-κB expression increased in GFP-Aire/DC compared with GFP/DC (Figure 4A). The IRF-3 and MyD88 expression levels were decreased in GFP-Aire/DC compared with the controls, even after Poly (I:C)treatment. TICAM-1 expression was only up-regulated in Poly (I:C)-treated GFP-Aire/DC. The Western blotting detection showed that in Aire-GFP/DC, the expression of NF-κB was increased compared with GFP/DC, but after being activated by Poly (I:C), decreased in Aire-GFP/DC. Phospho-NF-κB p65 was up-regulated in Aire-GFP/DC. IκB-α, as the inhibitor of NF-κB, expressed at a very low level in GFP-Aire/DC compared with GFP/DC, but increased in Poly (I:C)-treated GFP-Aire/DC, and the expression of phospho-IκB-α was increased in the GFP-Aire/DC, which may explain the regulated expression of cytokines. There was higher expression of IRF-3 in the control GFP/DC, and showed no difference after stimulation. The protein level of phospho-IRF-3 was un-detected using Western blotting (Figure 4B). Using FACS and immunofluorescence staining, we found the expression of phospho-NF-κB p65, phospho-IκB-α, and phospho-IRF-3 were all up-regulated in untreated and Poly (I:C)-treated Aire-GFP/DC (Figure S2A,B). Aire also enhanced NF-κB translocation to the nuclei, a process that might have promoted target gene transcription (Figure 4C). These data suggest that Aire might directly target NF-κB but not TICAM-1 or IRF-3 to up-regulate the production of cytokines in DC2.4 cells.

## 3. Discussion

DCs maturation correlates with the different functions of these cells. Immature DCs usually express low levels of co-stimulatory molecules, such as CD80 and CD86 and, thus, function as tolerogenic cells. In contrast, mature DCs induce efficient immune response via high levels of co-stimulatory molecules expression. Therefore, Aire might support the maintenance of immature DCs as a possible mechanism for maintaining peripheral immune tolerance. Additionally, eTAC were reported as tolerogenic cells characterized by the low CD80 and CD86 expression that could induce antigen-specific tolerance. TLR stimuli were not able to revert the eTAC-induced tolerance [6]. These reported features are similar to those of the Aire-expressing DC2.4 cells in our study. However, further studies will be required to define whether these cells represent the same population.

Our previous studies revealed that the TLR1, 3, and 8 expression levels were up-regulated in Aire-overexpressing murine macrophages and these macrophages polarized into M1. TLR3, as one of a number of key pattern recognition receptors, plays an important role in recognizing multiple pathogens, such as viruses and fungi, while the patients with Aire mutation usually suffered from chronic mucocutaneous candidiasis. In the current study, TLR3 up-regulation was also detected in Aire-overexpressing DC2.4 cells. This indicated that Aire might play an important function in defending *Candida* infection. Thus, we hypothesize that Aire can influence the function of DCs by regulating the expression of TLRs. It also reported after the activation of IRF-3 and NF-κB via TICAM-1 in the TLR3 pathway, following type I IFN and other cytokine production, which will affect the functions of DCs [14].

DCs promote the differentiation of naïve T cells into various T cell subsets via the production of different cytokines. For example, DCs secrete IL-27 and type I IFN via the TICAM-1 pathway after activing TLR3, and IL-27 supports Th1 polarization in early immune response. Type I IFN can also induce Th1 proliferation [3]. In contrast, other studies show that IL-27 could suppress the differentiation of Th1, Th2, and Th17, and promote the transformation of inflammatory T cells to IL-10, producing T cells. IL-27 stimulation can significantly improve Treg function both in vitro and in vivo. Type I IFN can also induce Treg phenotype polarization [15,16,17,18]. In this study, we observed the increases of IL-27 and IFN expression in untreated GFP-Aire/DC. These results might indicate that Aire in DCs can maintain self-tolerance by inducing Treg production. Following Poly (I:C) treatment, there were no significant differences in IL-27 and IFN expression between GFP-Aire/DC and GFP/DC; however, the increased fold of expression were lower in GFP-Aire/DC than those in GFP/DC, which indicated that Aire possibly can both take part in inflammatory response and regulate the immune tolerance through influencing the function of DCs.

Following ligand recognition, TLR3 is activated and plays a role in the initiation or regulation of downstream signaling and the activation of IRF-3 and NF-κB-dependent gene expression. TICAM (also known as the TIR domain-containing adaptor inducing IFN-β or TRIF), is a key adaptor molecule that links TLR3 engagement to transcriptional activation; TICAM is transiently recruited to endosomal TLR3 and indirectly activates several transcription factors, including IRF-3 and NF-κB, thus leading to the induction of type I IFN expression, cytokine/chemokine production and DC maturation via the expression of T cell stimulatory molecules (e.g., MHC class II, CD40, CD80, CD83, and CD86) [19]. IRF-3, a member of the IRF transcription factor family, is involved in TLR3 signal pathway-mediated transduction. Upon phosphorylation, IRF3 translocates into the nucleus and interacts with the transcriptional co-activator cAMP-responsive element binding protein (CBP)/p300 to induce a conformational change, thus exposing the IRF3 DNA binding domain and allowing the transcription of type I IFN [20,21]. CBP is the first identified partner of Aire [22] and was found co-localizing with Aire in nuclear bodies, synergizing Aire to initiate gene transcription. This indicates that Aire might competitively bind to CBP to impair IRF-3 activation. In some cells, such as mature B cells and macrophages, NF-κB can also be detected as a constitutively active nuclear protein [23,24]. In untreated cells, inactive NF-κB is retained in the cytoplasm with the inhibitor of nuclear factor κB (IκB). Upon TLR3 pathway activation, IκB is phosphorylated and degraded, thus allowing NF-κB to translocate into the nucleus where it binds to specific DNA sequence elements and activates gene transcription. NF-κB pathway activation can be down-regulated through several mechanisms. NF-κB can be shuttled back to the cytoplasm via IκB-α following the re-synthesis of IκB-α in an NF-κB-dependent manner after its degradation by IκB kinase (IKK). Additionally, the p65 NF-κB subunit binds to and is ubiquitinated by the nuclear protein PDZ and LIM domain protein 2 (PDLIM2) [23]. In the unstimulated cells of this study, Aire might regulate NF-κB expression and, thus, influence downstream genes, such as IL-12, IL-23, IL-27, and IFN-β. Upon Poly (I:C) stimulation, the TLR3 signaling pathway is triggered to activate NF-κB, which then translocates to the nucleus where it triggers down-regulatory mechanisms, thus resulting in reduced target gene expression.

## 4. Experimental Section

### 4.1. Cells and Animals

The DC2.4 cell line was obtained from the Southern Cell Company (Guangzhou, China), and the cells were cultured in RPMI-1640 (Gibco, Logan, UT, USA) supplemented with 10% heat-inactivated fetal bovine serum (FBS) (HyClone, Logan, UT, USA).

### 4.2. Cell Culture and Ligand Stimulation

Cells were plated in six-well culture plates (2 × 10^6^/well) overnight. The cells were subsequently washed twice in RPMI 1640 (Gibco) and stimulated. According to the results of preliminary experiments, the cells were exposed to 100 μg/mL of Poly (I:C) (Sigma, St. Louis, MO, USA) for 48 h and were then were harvested for real-time PCR, flow cytometry and Western blotting analysis.

### 4.3. Transfection

A pEGFPC1 vector that expressed the full-length murine Aire gene (pEGFPC1/Aire) was constructed as previously described [13]. The Lipofectamine™ 2000 Transfection Reagent (Invitrogen, Carlsbad, CA, USA) was used to transfect DC2.4 cells with either pEGFPC1/Aire or pEGFPC1 plasmids, according to the manufacturer’s protocol. Forty-eight hours later, the cells were subjected to selection with media containing 600 μg/mL of G418 (Amresco, Solon, OH, USA). After 14 days of culture in selection media, the G418-resistant clones were isolated. GFP signals from the transfected DC2.4 cells were determined via fluorescence microscopy (Olympus, Tokyo, Japan). The clones were further expanded and analyzed using flow cytometry, Western blotting, and immunofluorescence methods.

### 4.4. Quantitative Real-Time PCR (RT-qPCR)

RNAiso™ PLUS (Takara, Tokyo, Japan) was used to isolate total RNA from the cells. cDNA was synthesized from 1.0 μg of total RNA using reverse transcriptase M-MLV and oligo-dT according to the manufacturer’s instructions (Takara) in a total reaction volume of 20 μL. Oligonucleotide primers were designed with Primer Premier 5.0 (Table S1). Real-time PCR amplification of a cDNA template amount corresponding to 20 ng of total RNA was conducted on an ABI PRISM 7300 sequence detection system (Applied Biosystems, Foster, CA, USA) using SYBR Premix Ex Taq™ II (Takara) according to the manufacturer’s suggested protocol. The PCR conditions were 95 °C for 30 s and 40 cycles each comprising 15 s at 95 °C and 31 s at 60 °C, followed by a dissociation plot. For data analysis, the comparative threshold cycle (*C*_t_) value for GAPDH was used to normalize loading variations in the real-time PCR samples. The ΔΔ*C*_t_ value was then obtained by subtracting the control ΔΔ*C*_t_ values from the corresponding experimental Δ*C*_t_. The ΔΔ*C*_t_ values were converted to fold differences relative to the control with the equation 2^-^^ΔΔ*C*t^.

### 4.5. Flow Cytometry (FCM)

Cells were incubated with an FcR-blocking mAb (MACS, Teterow, Germany) first and subsequently fixed in 2% paraformaldehyde for 60 min at 4 °C. The cells were then centrifuged at 300× *g* for 5 min, after which the cell pellet was resuspended in 100 μL of 0.1% saponin (Sigma) to which 1 μg each of purified anti-TLR3, TLR7 and TLR8 rabbit polyclonal antibodies (Santa Cruz, Dallas, TX, USA) were added prior to a 40 min incubation at 4 °C. The cells were then washed and resuspended in 100 μL of 0.1% saponin, followed by incubation with 0.5 μg of PE-conjugated goat anti-rabbit IgG (Santa Cruz) for 40 min at 4 °C. The cells were washed with 0.1 M PBS (containing 0.1% saponin) and detected on a BD FACS Calibur flow cytometer (BD, San Jose, CA, USA).

For DC2.4 cell surface phenotype analysis, the cells were treated with or without Poly (I:C) for 48 h. The cells were subsequently harvested and incubated with an FcR-blocking mAb (MACS), followed by staining with mAb specific for CD40, CD80, CD83, CD86, CD11c, or MHC-II along with the corresponding isotype controls (eBiosciences, San Diego, CA, USA). All incubations were performed at 4 °C for 30 min. The cells were analyzed on a FACSCalibur flow cytometer (BD).

### 4.6. Western Blotting

The cells were harvested and the nuclear and cytoplasmic proteins were extracted as previously described [25]. The protein concentrations of all samples were measured using a BioSpectrometer (Eppendorf, Hamburg, Germany), and the samples were subjected to Western blotting overnight at 4 °C with the following antibodies: anti-β actin mouse monoclonal (Protein Tech, Rosemont, IL, USA), anti-Aire rabbit polyclonal, and anti-NF-κB p65 mouse monoclonal (Santa Cruz) at a 1:200 dilution; anti-IκB-α,anti-IRF-3 (Abcam, Cambridge, MA, USA), anti-phospho-NF-κB p65, and anti-phospho-IRF-3 rabbit monoclonal (CST, Beverly, MA, USA) at a 1:1000 dilution; and an anti-phospho (Ps36) IκB-α rabbit monoclonal (Abcam) at a 1:10,000 dilution. The membranes were probed with secondary antibodies (goat anti-rabbit IgG/HRP and goat anti-mouse IgG/HRP, 1:2000 dilution; Protein Tech) for 1.5 h at 37 °C. Protein expression was detected with a GeneGnome Imaging System (Gene Company, Hong Kong, China) and Western Lightning Plus-ECL (PerkinElmer, Fremont, CA, USA).

### 4.7. Immunofluorescence Staining

The stably pEGFPC1/Aire or pEGFPC1-transfected DC2.4 cells were seeded into a 96-well plate and subjected to immunofluorescence labeling with a Cellular NF-κB Translocation Kit (Beyotime Biotech, Nanjing, China) according to the manufacturer’s instructions. Briefly, after washing and fixing, the cells were incubated with a blocking buffer for 1 h to suppress non-specific binding. Next, the cells were incubated with the primary NF-κB p65 antibody for 1 h at 37 °C, followed by incubation with a Cy3-conjugated secondary antibody for 1 h at 37 °C and DAPI for 5 min. The fluorescence signals were observed with a fluorescence microscope (Olympus, Tokyo, Japan).

### 4.8. ELISA

Culture supernatants were analyzed via sandwich ELISA with ELISA kits (eBioscience, USA) specific for mouse IL-12p40, IL-23, IL-27p28, or IFN-β, according to the manufacturer’s recommendations. The cytokine concentrations in the supernatants were calculated according to standard curves (BioTek, Burlington, VT, USA).

### 4.9. Statistical Analysis

All statistical analyses are expressed as the means ± standard deviations (SD) from at least three samples per data point. Student’s *t*-test was used to determine the significance of the results (significance: * *p* < 0.05; ** *p* < 0.01).

## 5. Conclusions

In summary, the data revealed that, in DC2.4 cells, Aire expression up-regulated the TLR3 expression and influenced key molecules, such as NF-κB in the TLR3 pathway to target the downstream genes. These findings suggest that Aire might contribute to regulating inflammatory responses, as well as tolerance, through influencing TLR3 and cytokines downstream of its pathway.

## Figures and Tables

**Table 1:** The effects of Aire on the Poly (I:C)-stimulated phenotypic characteristics of DC2.4 cells.

MFI %	GFP/DC		GFP-Aire/DC	
	Control	Poly (I:C)	Control	Poly (I:C)
CD40	0.50 ± 0.25	2.60 ± 0.18 ^1^	0.64 ± 0.22	0.98 ± 0.27 ^2,3^
	1.67 ± 0.64	8.35 ± 0.35 ^2^	6.61 ± 0.13	7.34 ± 1.02
CD80	51.60 ± 12.16	69.60 ± 0.64	26.20 ± 8.76 ^2^	14.51 ± 10.57 ^3^
	30.43 ± 7.36	43.67 ± 1.37	25.99 ± 0.02	8.08 ± 4.57 ^4^
CD83	17.07 ± 0.49	8.46 ± 0.85 ^1^	13.60 ± 1.70	4.08 ± 3.29 ^3^
	13.75 ± 3.17	13.18 ± 9.03	11.75 ± 1.27	9.02 ± 3.89
CD11C	7.93 ± 0.49	11.70 ± 0.28 ^2^	6.69 ± 0.22	5.03 ± 0.18 ^4^
	15.59 ± 5.51	19.60 ± 0.87	17.41 ± 2.75	16.14 ± 0.20
CD86	1.82 ± 0.39	2.70 ± 1.00 ^2^	2.89 ± 0.25 ^2^	2.34 ± 0.19 ^2^
	4.03 ± 0.38	5.88 ± 1.75 ^2^	3.59 ± 0.05 ^2^	6.60 ± 0.68
MHC II	0.29 ± 0.14	1.09 ± 0.11 ^2^	0.42 ± 0.29	0.42 ± 0.16 ^3^
	0.62 ± 0.41	1.37 ± 0.43	2.42 ± 0.01 ^1^	2.22 ± 1.93

GFP/DC and GFP-Aire/DC were treated with Poly (I:C) for 48 h as described in the Methods. Subsequently, the phenotypic characteristics of GFP/DC or GFP-Aire/DC in the presence or absence of Poly (I:C) treatment were determined by FCM. The results are shown as the means ± SD from three to six independent experiments. %, percentage of positive cells; MFI, mean fluorescence intensity. ^1^ *p* < 0.01 and ^2^ *p* < 0.05 compared with control GFP/DC; ^3^ *p* < 0.05 and ^4^ *p* < 0.01 compared with Poly (I:C)-treated GFP/DC.

## Supplementary Materials

Supplementary materials can be found at www.mdpi.com/1422-0067/17/12/2002/s1.
