# Supplementary material for: Autoimmune Regulator Expression in DC2.4 Cells Regulates the NF-κB Signaling and Cytokine Expression of the Toll-Like Receptor 3 Pathway
Source: Int J Mol Sci. 2016 Dec 1;17(12):2002. doi: 10.3390/ijms17122002 (PMC5187802; doi:10.3390/ijms17122002)
Supplement: Supplementary file 1 [file ijms-17-02002-s001.pdf]

Supplementary Material: Autoimmune Regulator Expression in DC2.4 Cells Regulates the NF-κB Signaling and Cytokine Expression of the Toll-Like Receptor 3 Pathway

Jitong Sun, Kunwei Niu, Haiying Fu, Haijun Li, Yi Li and Wei Yang

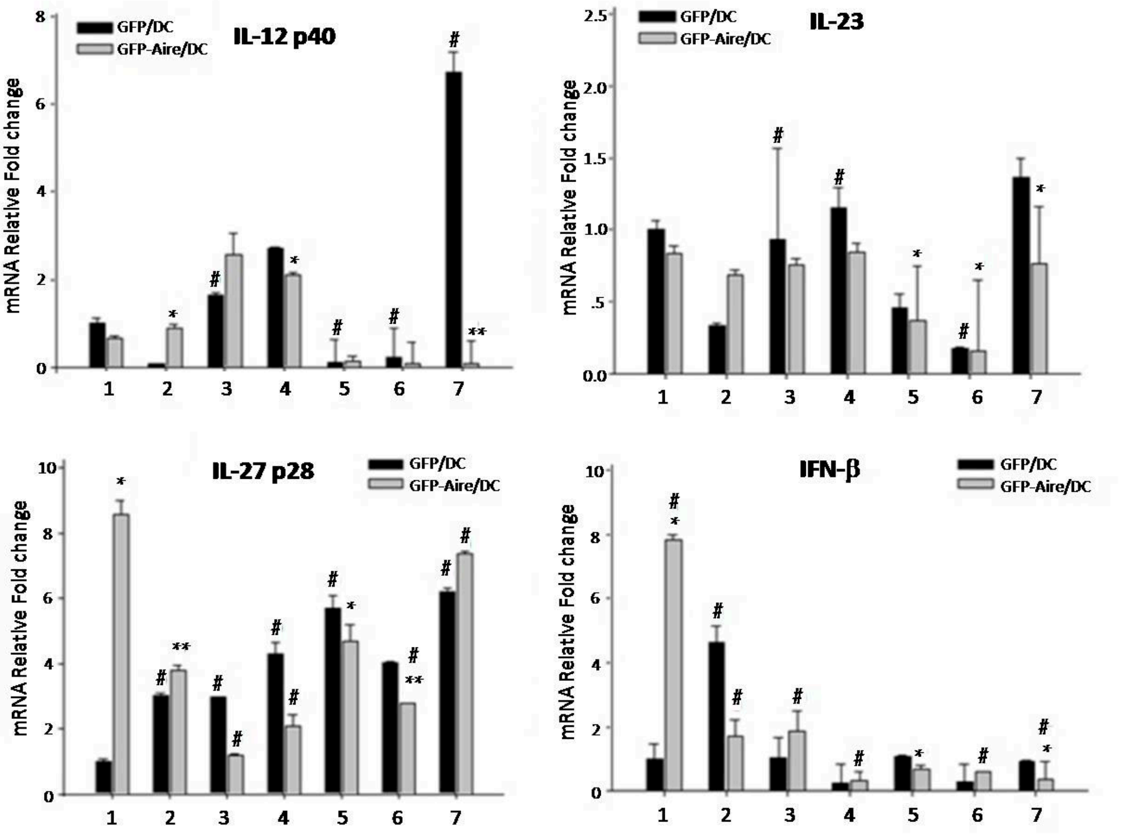

Figure S1. Effects of Aire on downstream gene expression in GFP-Aire/DC cells treated with a lower dose of Poly (I:C). The levels of IL-12 p40, IL-23, IL-27 p28, and IFN-β transcript expression in GFP-Aire/DC and GFP/DC cells were detected by RT-qPCR. All qPCR data are shown as the gene expression relative to GAPDH expression and are depicted as the fold change relative to GFP/DC cells, which were normalized to 1. *x*-axis: (**1**): Control; (**2**,**3**): 3 h Poly I:C at 1 and 12.5 mg/mL, respectively; (**4**,**5**): 6 h Poly I:C at 1 and 12.5 mg/mL, respectively; (**6**,**7**): 12 h Poly I:C at 1 and 12.5 mg/mL, respectively. * $p < 0.05$, ** $p < 0.01$ compared with the GFP/DC control; # $p < 0.05$ compared with the GFP-Aire/DC control.

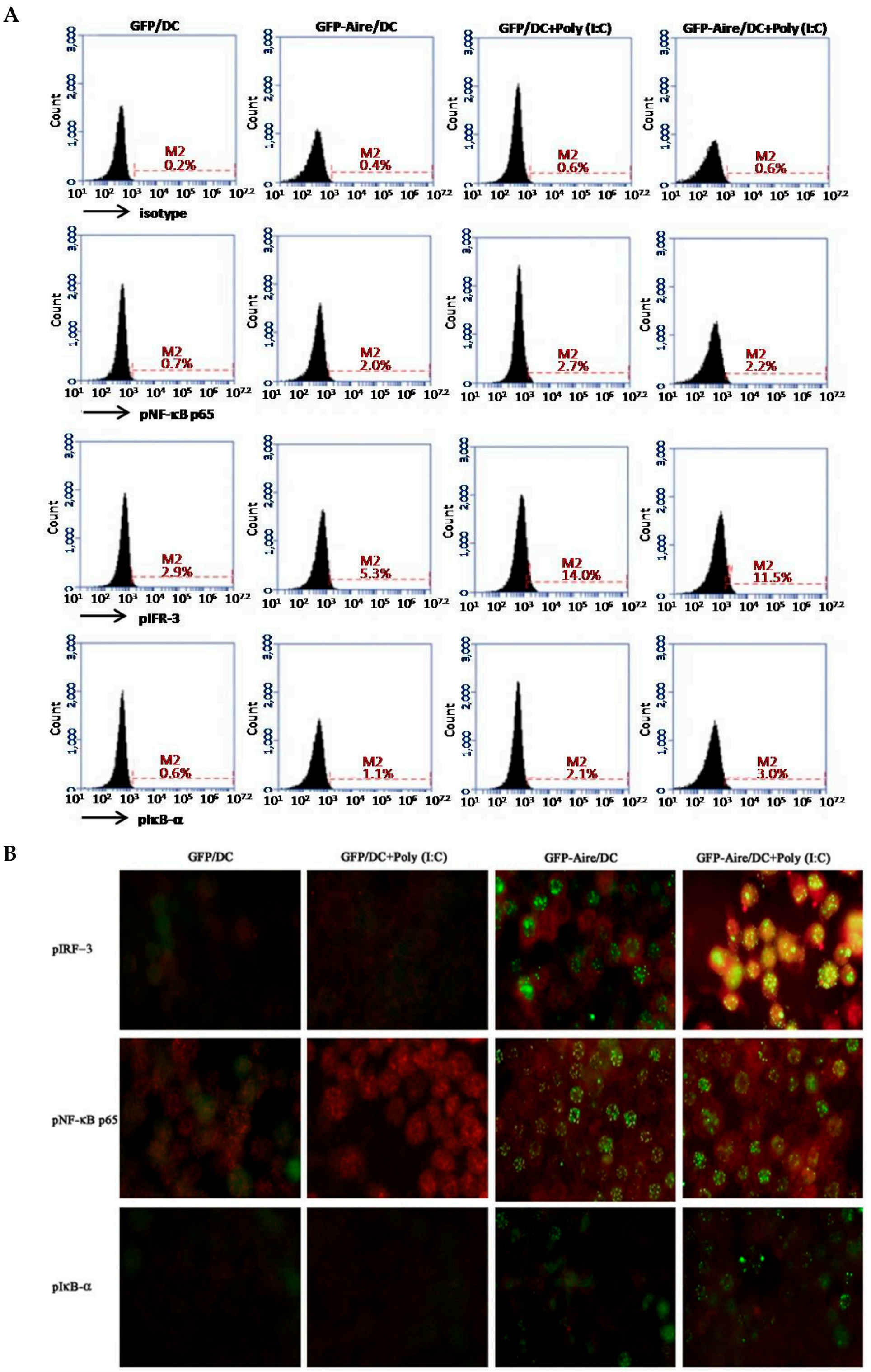

Figure S2. (**A**) Immunofluorescent detection of NF-κB, IRF-3, and IκB-α (**red**) in GFP/DC and GFP-Aire/DC treated with Poly (I:C) via confocal microscopy. Original magnification: 200×; (**B**) Phospho-NF-κB, IκB-α, and IRF-3 expression were detected by FCM. The results are representative of at least three independent experiments.

Table S1. Oligonucleotide primer sequences.

Gene	Sequences	Size (bp)
GAPDH	S: 5′-GACTTCAACAGCAACTCCCACTC-3′<br>AS: 5′-TAGCCGTATTCATTGTCATACCAG-3′	107
TLR1	S: 5′-CTGAGGGTCCTGATAATGTCCTAC-3′<br>AS: 5′-GATCACCTTTAGCTCATTGTGGG-3′	114
TLR2	S: 5′-TTGCGTTACATCTTGGAACTG-3′<br>AS: 5′-ACTACGTCTGACTCCGAGGG-3′	96
TLR3	S: 5′-CAACGGTTCCTTCTCCTATCTC-3′<br>AS: 5′-TTGCTTAGTAAATGCTCGCTTC-3′	139
TLR4	S: 5′-CTTCATTCAAGACCAAGCCTTTC-3′<br>AS: 5′-AACCGATGGACGTGTAAACCAG-3′	126
TLR5	S: 5′-TCTACAACATATCCACCGAAGACTG-3′<br>AS: 5′-TTATGACTACAAGGGTGATGACGAG-3′	123
TLR6	S: 5′-ACAACATTCCCAGTAGATACCACA-3′<br>AS: 5′-TCCTCATTGACTAAGGCTAACTTC-3′	148
TLR7	S: 5′-TGAGGGCATTCCCACTAACAC-3′<br>AS: 5′-TCCAGATGGTTCAGCCTACGG-3′	96
TLR8	S: 5′-TTCCTCACATTCCTTACCACCTC-3′<br>AS: 5′-GTGATAGATAAACCAAACATCCCAG-3′	87
TLR9	S: 5′-TCTGTCTTACTACACCGCTATTTG-3′<br>AS: 5′-AAACTACCCTTTACAGCCAACC-3′	93
IL-12p40	S: 5′-ACTGGAACTACACAAGAACGAGAG-3′<br>AS: 5′-CTTCAAGTCCTCATAGATGCTACCA-3′	108
IL-23	S: 5′-AAATAATGTGCCCCGTATCCAGT-3′<br>AS: 5′-TCCCCTTTGAAGATGTCAGAGTC-3′	140
IL-27p28	S: 5′-CTCTGCTTCCTCGCTACCAC-3′<br>AS: 5′-GGGGCAGCTTCTTTTCTTCT-3′	234
IFN-β	S: 5′-AAGAGTTACACTGCCTTTGCCATC-3′<br>AS: 5′-CACTGTCTGCTGGTGGAGTTCATC-3′	135
MyD88	S: 5′-TCCGGCAACTAGAACAGACAGACT-3′<br>AS: 5′-GCGGCGACACCTTTTCTCAAT-3′	119
NF-κB	S: 5′-GGACCTATGAGACCTTCAAGAG-3′<br>AS: 5′-CTACCCGAAACTCAACTTCTGT-3′	111
TICAM	S: 5′-GATGGAGAGGTCCACAAGGA-3′<br>AS: 5′-GAGTGTAGCGTGGGGAGTGT-3′	109
IRF-3	S: 5′-AGCCTGCATCAAATCAACC-3′<br>AS: 5′-CCACCTTTCTGGCGAAGA-3′	180
